# Editorial: Herpesvirus Maturation

**DOI:** 10.3389/fmicb.2020.00657

**Published:** 2020-04-15

**Authors:** Ritesh Tandon, Jens von Einem

**Affiliations:** ^1^Department of Microbiology and Immunology, The University of Mississippi Medical Center, Jackson, MS, United States; ^2^Institute of Virology, Ulm University Medical Center, Ulm, Germany

**Keywords:** herpes, CMV, maturation, virus, virus assembly, nuclear replication, cytoplasmic maturation

All three subfamilies (α, β, and γ) of herpesviruses undergo a maturation phase during replication that initiates in the nucleus of the infected cell with encapsidation of viral DNA to form nucleocapsids. These nucleocapsids are transported across the nuclear membrane into the cytoplasm utilizing a sophisticated nuclear egress complex (NEC). Cytoplasmic phase of virus maturation includes tegumentation and envelopment of nucleocapsids. Herpesvirus tegument proteins play important roles in maintaining the stability of capsids and directing the acquisition of virus envelope. The maturation concludes with the envelopment of nucleocapsids, which occurs at modified host membranes in the cytoplasm and exploits host vesicular trafficking. The entire process of virus maturation is orchestrated by protein-protein interactions and enzymatic activities of virus and host origin.

Herpesvirus maturation remains one of the least studied yet most complicated part of virus life cycle and numerous host and viral factors appear to influence the outcome. The current collection contains three articles contributed by nine authors. These articles have already gathered nearly 5,000 views and continue to gather attention from scientists and non-scientists alike who are willing to delve deep into the mysteries of herpesvirus maturation. The first article by Close et al. focuses on the contribution of endocytic recycling compartment to human cytomegalovirus (HCMV) maturation and egress. They make a strong case that cellular transport systems are engaged by HCMV in a systemic manner for successful maturation and egress. They utilize tools in advanced transcriptomics and proteomics married with computational analysis to come to these conclusions. Earlier studies by the same group as well as other groups have alluded to this engagement process. Close et al. argue that the composition and behavior of endosecretory organelles change during the biogenesis of cytoplasmic virion assembly complex (referred to as cVAC, vAC, or AC in literature). Infection-associated changes in gene expression suggest shifts in the balance between endocytic and exocytic recycling pathways, leading to the formation of a secretory trap within the cVAC. Also, a shift toward outbound secretory vesicle trafficking indicate a potential role of cVAC in virion egress. The analysis of signaling pathways lead them to model the behavior of endocycling recycling compartment (ERC) during HCMV replication.

The article by Grosche et al. discusses HSV-1 replication in monocyte-derived dendritic cells (DCs). HSV-1 completes its gene expression profile in immature as well as mature DCs; however, lytic infection only occurs in immature DCs and mature DCs fail to release infectious progeny into the supernatant pointing toward differences in virus maturation and release. This article provides a commentary on viral as well as host factors responsible for these differences.

The article by Heilingloh and Krawczyk focuses on the significance of production of non-infectious L-particles during HSV infection and discusses their biological functions. These particles are mostly composed of viral tegument proteins and are devoid of viral DNA and capsids. L-particles seem to undergo similar maturation and egress as the infectious particles and their generation is therefore interesting to investigate in order to understand the process of virus maturation.

We believe that maturation of herpesviruses will be an area of continued interest in virology as new virus and host players continue to emerge. The dramatic reorganization of the host endosecretory system leading to the formation of cVAC has mainly been appreciated in HCMV infected cells and deemed as the site of virion assembly. The debate is still on the floor whether this cVAC is a functional component of the virus maturation pathway or is merely an after-effect of virus replication. Although a typical cVAC is not seen during infection with other herpesviruses, a perinuclear site of virus maturation in cytoplasm has been recognized in most. Future studies in this area should focus on the identification of functional interactions among host and viral factors that determine virus maturation as well as structural characterization of the site of virus maturation in cells using advanced techniques in fluorescence and electron microscopy.

## Author Contributions

All authors listed have made a substantial, direct and intellectual contribution to the work, and approved it for publication.

### Conflict of Interest

The authors declare that the research was conducted in the absence of any commercial or financial relationships that could be construed as a potential conflict of interest.

